# Utility of Continuous Glucose Monitoring in the Management of a Suicide Attempt in a Patient With Comorbid Type 2 Diabetes and Major Depressive Disorder

**DOI:** 10.7759/cureus.107040

**Published:** 2026-04-14

**Authors:** Francisco José Barbosa-Camacho, Roberto Carlos Miranda-Ackerman, Javier Contreras-Cárdenas, Sergio Armando Covarrubias-Castillo

**Affiliations:** 1 Psychiatry, Antiguo Hospital Civil de Guadalajara "Fray Antonio Alcalde", Guadalajara, MEX; 2 Critical Care Medicine, Hospital San Javier, Guadalajara, MEX; 3 Psychiatry, Hospital Psiquiátrico Fray Bernardino Álvarez, Mexico City, MEX; 4 Medical Clinics, Centro Universitario de Ciencias de la Salud, Universidad de Guadalajara, Guadalajara, MEX

**Keywords:** borderline personality disorder, continuous glucose monitoring, insulin overdose, suicide prevention, type 2 diabetes

## Abstract

Type 2 diabetes mellitus is a highly prevalent chronic disease. In patients with comorbid psychiatric disorders, insulin may become a means of suicide due to its potentially lethal effects when overdosed. Continuous glucose monitoring (CGM) systems have proven effective for glycemic control, but their role in suicide prevention is less explored. We report the case of a 68-year-old woman with type 2 diabetes, borderline personality disorder, and major depressive disorder, who attempted suicide by injecting 90 units of lispro insulin and ingesting 12 tablets of lorazepam. The patient's daughter, monitoring her mother’s glucose levels via CGM, detected a sudden hypoglycemic drop and intervened promptly, preventing a fatal outcome. The patient was treated in the emergency department with intravenous dextrose and supportive care, was later admitted to the ICU, and followed by psychiatric evaluation and pharmacological adjustments. Her psychiatric treatment plan was modified to reduce central nervous system depressants and reinforce outpatient psychiatric follow-up. At a one-month follow-up, the patient reported clinical improvement, denied suicidal ideation, and remained under psychiatric care. This case underscores the utility of CGM not only for glycemic control but also as an emergency detection tool in high-risk psychiatric patients, emphasizing the value of integrating digital health with psychiatric monitoring and family support.

## Introduction

Type 2 diabetes mellitus (T2DM) is a highly prevalent chronic condition in Latin America, with Mexico reporting an ever-increasing prevalence, reaching 16.9% as of 2021, according to the International Diabetes Federation [1]. Many patients require insulin therapy, which, while lifesaving, can become a tool for self-harm in vulnerable individuals [2,3].

The treatment of choice for some patients is often the subcutaneous administration of insulin, due to its similarity to the biologically produced insulin in the human body. Patients with T2DM typically monitor their blood glucose levels before insulin administration using a glucose sensor. This involves a fingerstick to obtain a blood sample, which is then collected on a test strip and read by the sensor. Although this practice may appear harmless when performed occasionally, it can become problematic for patients who perform it daily, leading to pain, discomfort, and reduced adherence to monitoring recommendations [4]. In recent years, continuous glucose monitoring (CGM) systems have used a subcutaneous sensor to monitor serum glucose levels continuously, offering real-time data and alerts and eliminating the need for fingersticks [4,5]. Systematic reviews have demonstrated that CGM implementation in primary care settings provides significant clinical benefits, including improved glycemic control, enhanced patient engagement, and timely identification of glucose excursions that would otherwise remain undetected with conventional monitoring [5]. Recent meta-analytic evidence reported that the use of CGM was associated with better management of glycemic parameters and lower HbA1c levels, as well as a lower range of hypoglycemia events and glycemic variability during the day in comparison to traditional self-monitoring of blood glucose. This could translate into more stable control throughout the day and night, adding a layer of safety, particularly in patients at risk for hypoglycemic events or intentional insulin misuse [6].

Their integration into family-centered care offers remote monitoring capabilities that enable data sharing with family members, caregivers, and healthcare providers in real time through secure cloud-based platforms and mobile applications. This functionality allows for immediate notification of concerning glucose patterns or critical events, such as severe hypoglycemia, even when patients are alone, asleep, or otherwise unable to respond. The combination of continuous data availability and remote accessibility fundamentally transforms diabetes management from episodic self-assessment to comprehensive, proactive monitoring [7].

Major depressive disorder (MDD) is one of the leading causes of disability worldwide, affecting approximately 3-5% of adults globally [8,9]. However, the Latin American population exhibits especially higher prevalence, in some reports as high as 12% [10] and reaching almost 25% in some Mexican samples [11]. MDD is notably prevalent among individuals with T2DM [12-15], and its presence can exacerbate the challenges of diabetes management, leading to poorer glycemic control and increased complications. Some authors have studied the bilateral relationship between T2DM and mood disorders, reporting that the odds of presenting a depressive disorder can be as high as 1.8 to 2.2 times more likely in patients with T2DM when compared with non-diabetic controls [9]. Accordingly, the risk of suicide in patients with T2DM is significantly elevated [14]. One concerning method of suicide among patients with diabetes is insulin overdose. Insulin, while lifesaving when used appropriately, can be lethal in excessive amounts. An intentional overdose can result in severe hypoglycemia, leading to neurological injury, coma, and potentially death. However, determining a universally lethal dose is challenging due to individual variations in insulin sensitivity, body weight, and overall health status [16]. It has been shown that suicides using insulin among insulin-dependent diabetics are equally as prevalent, if not more so, than fatal accidental insulin overdoses.

We present the case of a woman with borderline personality disorder (BPD), MDD, and T2DM who attempted suicide by combining a high dose of insulin and lorazepam. Timely recognition of an abrupt glucose decline by her daughter -- via the CGM device -- enabled prompt medical attention and likely prevented a fatal outcome. This case highlights the potential of CGM not only in metabolic control but also in the early detection of suicide attempts involving insulin.

## Case presentation

This is a 68-year-old female patient with a long-standing history of MDD and BPD, currently treated with duloxetine, quetiapine, lorazepam, pregabalin, and pramipexole. The patient has a history of five previous suicide attempts, all with warning signs and high lethality, requiring hospitalization in general medical units. However, none of these events resulted in psychiatric inpatient admission; she attended only sporadic outpatient follow-up with a private psychiatrist. She was also diagnosed with T2DM, managed with insulin glargine and lispro, and had a prior ischemic stroke of unspecified etiology in 2017.

Her current pharmacological regimen includes multiple central nervous system active agents, such as morphine and pramipexole, whose use and indications were unknown to the family, raising concerns about unsupervised polypharmacy. In addition to psychiatric and antidiabetic agents, she was prescribed medications for hypertension (losartan), hyperlipidemia (atorvastatin), gastrointestinal protection (omeprazole), urinary symptoms (mirabegron), and various nutritional supplements (folic acid, multivitamins, vitamin D, and monthly injectable complex B formulations) (Table 1). She uses a CGM device that transmits real-time data to her relatives' mobile devices.

**Table 1:** Pharmacological regimen and relevance to clinical risk profile The concurrent use of morphine, pregabalin, lorazepam, and quetiapine represents a significant sedative burden, compounding the risk of respiratory depression and potentially masking the clinical presentation of hypoglycemia. The indication for morphine and pramipexole was unknown to the patient's family, raising concerns about unsupervised polypharmacy. CNS, central nervous system; SNRI, serotonin-norepinephrine reuptake inhibitor.

Drug	Class	Relevance to suicide/clinical risk
Duloxetine	SNRI antidepressant	Potential for overdose; requires monitoring in high-risk patients
Quetiapine	Atypical antipsychotic	CNS depressant; sedative burden; overdose risk
Lorazepam	Benzodiazepine	Used in a suicide attempt; high lethality in combination with other CNS depressants
Pregabalin	Gabapentinoid	CNS depressant; misuse potential; potentiates sedation
Pramipexole	Dopamine agonist	Associated with impulse control disorders; indication unclear
Morphine	Opioid analgesic	High sedative burden; indication unknown to family; potentiates respiratory depression
Insulin glargine	Basal insulin	Lethal in overdose; requires supervised access in high-risk patients
Insulin lispro	Rapid-acting insulin	Used in a suicide attempt; directly responsible for a hypoglycemic crisis

**Figure 1:**
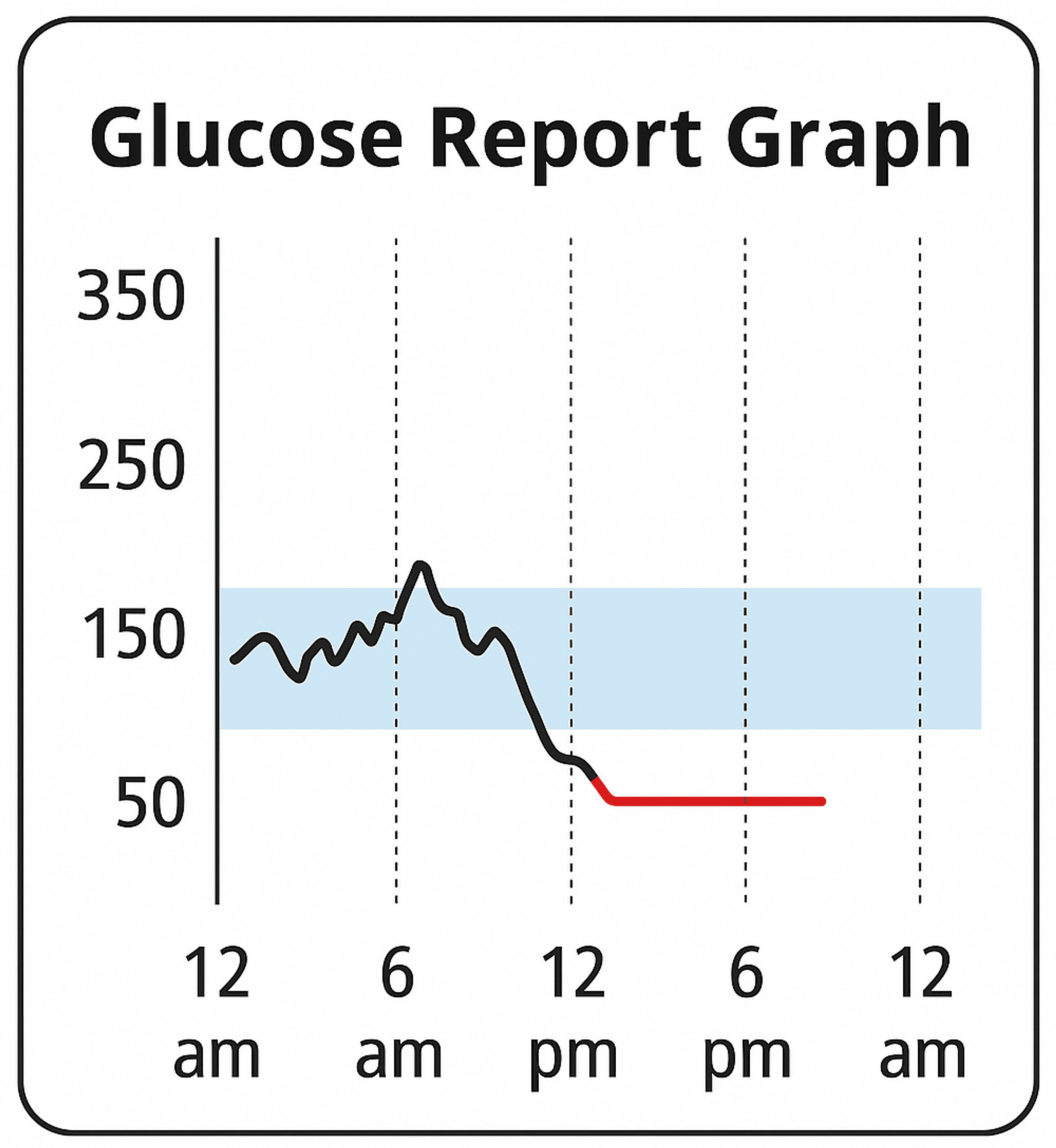
CGM graphic report CGM, continuous glucose monitoring.

**Figure 2:**
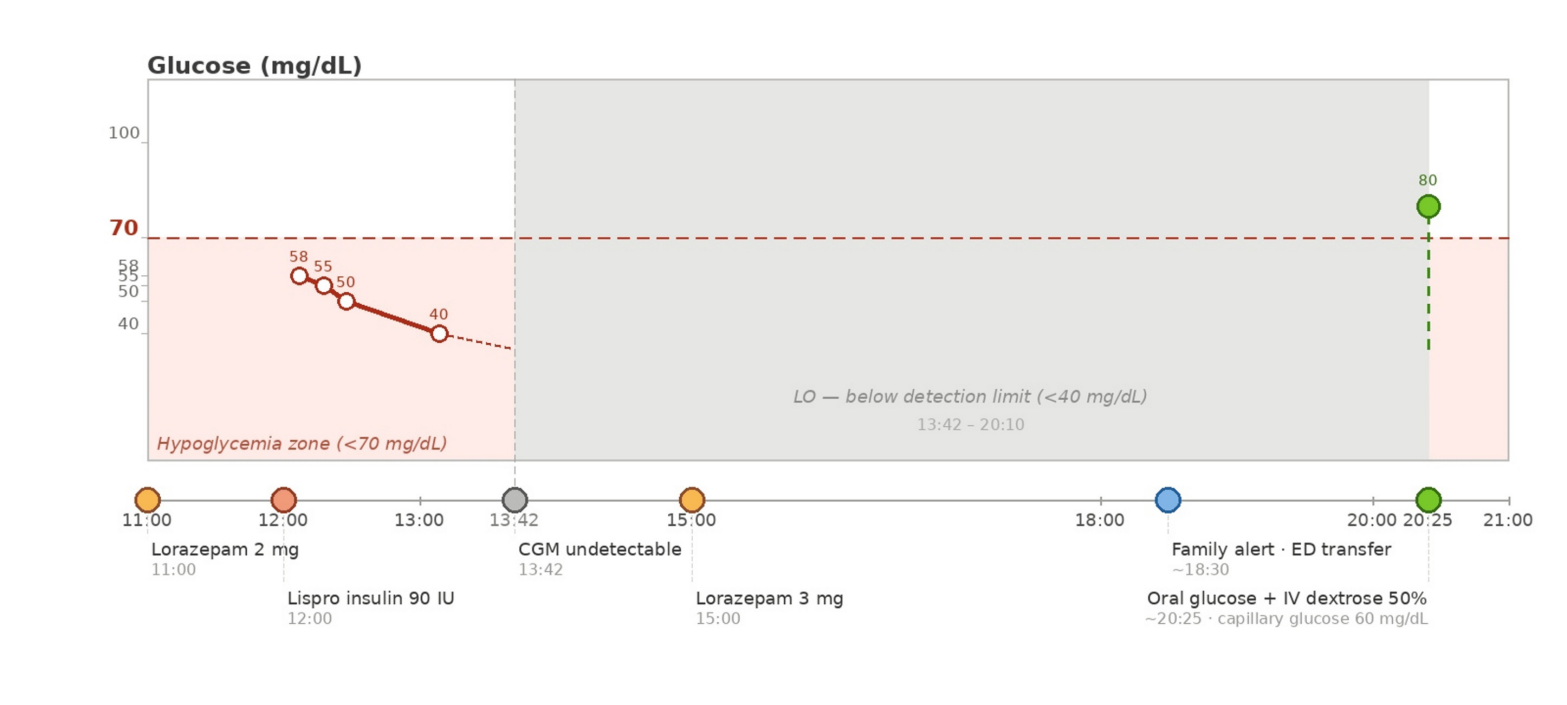
CGM readings and clinical event timeline (September 6) Shaded red zone: hypoglycemia (<70 mg/dL). Gray zone: CGM signal below detection threshold (<40 mg/dL, displayed as "LO" by device). CGM data are reported as measured by the device log. Capillary glucose at ED arrival: 60 mg/dL (obtained after oral glucose administration by family). Colored markers on the time axis indicate: orange = lorazepam dose; red-orange = insulin overdose; gray = CGM threshold event; blue = family intervention; green = treatment/ED arrival. CGM, continuous glucose monitoring; ED, emergency department; IU, international units; IV, intravenous.

On the day of the incident, the patient was brought to the emergency department by paramedics after being found somnolent at home by her daughter. CGM data revealed that glucose levels had already dropped into the hypoglycemic range as early as 12:07 p.m. (within minutes of the insulin injection), reaching 40 mg/dL by 1:09 p.m. and falling below the device's detection threshold (<40 mg/dL) by 1:42 p.m. The patient's daughter noticed the alarm on the CGM mobile application at approximately 6:30 p.m. and, upon arriving at the patient's home, found her somnolent and lying next to a partially empty blister pack of lorazepam (Figures 1, 2). According to the patient, she had experienced suicidal ideation earlier that morning and had proceeded to inject 90 units of lispro insulin and ingest 12 tablets of lorazepam 5 mg in a suicide attempt.

The patient's daughter provided her with two teaspoons of sugar, one teaspoon of honey, and a can of cola. Noticing that her drowsiness did not improve, they decided to transport her to the emergency department of a general hospital. Upon arrival, the patient was found to be somnolent but oriented, with a strong cough reflex. She was irritable and showed minimal cooperation with hospital personnel. On arrival, vital signs were as follows: blood pressure 116/50 mmHg, heart rate 100 bpm, respiratory rate 19 breaths/min, and temperature 35.6°C. Anthropometric data included a height of 168 cm and a weight of 70 kg (BMI: 24.8 kg/m²). A psychiatric assessment was conducted shortly after intravenous administration of 50 mL of 50% glucose solution and vital signs stabilization. The patient's somnolence at presentation likely reflected a combined effect of profound hypoglycemia and central nervous system depression secondary to lorazepam ingestion. Distinguishing the relative contribution of each factor was not possible clinically; however, the rapid improvement in consciousness following intravenous dextrose administration suggests hypoglycemia as the predominant driver.

Presenting symptoms

According to family members, during the preceding week, the patient had exhibited marked emotional lability, increased irritability, and worsening depressive symptoms, culminating in the suicidal act on the day of admission.

Mental status examination

We performed a mental status examination that found the patient slightly disheveled, wearing her own clothing underneath the hospital gown. She was irritable and minimally cooperative with hospital staff. Her speech was reduced in rate, volume, and spontaneity; her mood was dysphoric, while her affect appeared constricted and congruent with a depressed mood, stating 'Why are you helping me? I just want to die' (sic). Her thought process appeared tangential, with pervasive ideas of guilt, hopelessness, and worthlessness. She denied active suicidal ideation at the time of assessment; however, she acknowledged passive death wishes and confirmed that the ingestion was carried out with suicidal intent. Insight into her psychiatric condition was absent. She demonstrated poor impulse control and low frustration tolerance.

After vital signs stabilization, the patient was admitted to the intensive care unit (ICU).

Diagnostic assessment

The initial assessment was clinical and based on the patient’s presentation, psychiatric history, and collateral information provided by family members. The diagnosis of BPD and MDD, recurrent episode, severity, and suicidal behavior was confirmed according to the Diagnostic and Statistical Manual of Mental Disorders, Fifth Edition (DSM-5) criteria. The SAD PERSONS scale was applied, yielding a score of 5/10 (points assigned for age >45 years, history of depression, previous suicide attempts, widowed status, and chronic medical illness), consistent with moderate suicide risk and indicating the need for close psychiatric follow-up and structured outpatient care. 

From a medical standpoint, hypoglycemia was confirmed upon arrival with a bedside capillary glucose measurement of 60 mg/dL, obtained after oral glucose administration by the patient's daughter before arrival. The pre-intervention glucose value was not independently documented in the clinical record, which limits precise correlation between the CGM trace and clinical severity at the time of discovery. The patient's condition improved following intravenous administration of 50 mL of 50% dextrose solution. No diagnostic challenges were identified, and no alternate differential diagnoses were considered at the time, given the clear intentional overdose and psychiatric background.

Therapeutic intervention

Upon arrival, the patient was administered 50 mL of 50% dextrose solution intravenously, with prompt recovery of consciousness. Her vital signs stabilized, and she was admitted to the ICU for 48 hours of close monitoring, with no further episodes of hypoglycemia or respiratory depression observed.

During hospitalization, a comprehensive pharmacological review was conducted. Given the significant sedative burden identified in the patient's regimen (particularly the concurrent use of morphine, pregabalin, lorazepam, and quetiapine), a reassessment of potential drug interactions and their contribution to clinical instability was prioritized. Psychiatric consultation led to adjustments aimed at improving mood stabilization and minimizing CNS depressant load. The use of morphine and pramipexole was flagged for re-evaluation by her primary care and neurology teams. A plan was initiated for inpatient psychiatric admission following discharge, with the aim of establishing a structured pharmacological and psychotherapeutic treatment framework under close supervision.

Follow-up and outcomes

After two days in the ICU, the patient remained clinically stable and was discharged to a psychiatric facility for continued inpatient care. However, a follow-up emergency room visit one month later revealed that the patient had not adhered to the planned psychiatric hospitalization. She presented with a superficial laceration sustained while gardening, which she described as accidental; she explicitly denied suicidal ideation or intent upon directed psychiatric reassessment.

During that evaluation, the patient reported attending only private outpatient psychiatric consultations. She described subjective clinical improvement, with greater emotional stability and reduced impulsivity. She maintained the use of her continuous glucose monitor and had experienced no further hypoglycemic episodes or suicide attempts.

## Discussion

This case highlights the complex intersection between chronic illness, psychiatric comorbidity, and the use of emerging technologies as potential life-saving tools. While subcutaneous insulin remains a cornerstone in the treatment of T2DM, its accessibility in patients with comorbid psychiatric disorders represents a unique clinical challenge. In this case, the patient’s BPD, characterized by emotional dysregulation, impulsivity, and a history of major depressive episodes, created a high-risk scenario in which access to insulin became a means for self-harm.

Suicide risk in patients with diabetes, particularly those with comorbid mood or personality disorders, is significantly elevated. Several studies have shown that individuals with diabetes have up to two to three times the risk of depression compared with the general population, and when depression is present, the risk of suicidal behavior is markedly increased​ [16]. A systematic review by Assad et al. found that insulin overdose, although not a common method, has been used in both diabetic and non-diabetic individuals as a method of suicide, with subcutaneous injections being the most frequent route​. While insulin overdose can lead to profound hypoglycemia, coma, and death, there is no clearly defined lethal dose due to inter-individual variation in insulin sensitivity, weight, liver function, and glycemic baseline​ [13]. Importantly, up to 90% of patients admitted to toxicology units for insulin overdose report suicidal intent​, underscoring the need for proactive mental health screening in this population.

The intersection of T2DM and BPD intensifies this risk profile, with emerging data suggesting greater lethality in suicide attempts within this subgroup. This increased lethality is hypothesized to stem from the characteristic impulsiveness of BPD, which is associated with the use of more violent or definitive methods of self-injury. Moreover, the co-occurrence of depressive and anxious symptoms within BPD further compounds this vulnerability, underscoring the critical need for integrative, multidisciplinary care [14,17-19]. The psychological load of living with chronic illness, combined with unstable interpersonal functioning and affective instability, creates a scenario in which access to a pharmacologic agent such as insulin, readily available and medically sanctioned, may circumvent typical safeguards against impulsive suicidal action.

What distinguishes this case is the crucial role of a CGM system, which alerted the patient's relatives to a sudden and dangerous drop in glycemia. CGM technology, increasingly used in diabetes management, provides real-time feedback on glucose trends and has been shown to improve glycemic control and reduce hypoglycemic events. However, its potential role as a psychiatric early-warning system in high-risk patients has been largely unexplored. To our knowledge, only one prior case has documented a deliberate insulin overdose captured on CGM [20]. In that case, a blinded CGM system failed to detect severe hypoglycemia during a 300-unit insulin aspart overdose, with a mean absolute relative difference of 52% in the hypoglycemic range, underscoring the limitations of non-real-time monitoring. In contrast, our case involves a consumer-grade real-time CGM with active family data sharing, in which the device's remote alert function, rather than retrospective tracing, served as the life-saving mechanism. This distinction highlights a fundamentally different and underexplored use scenario: CGM not as a passive recorder of a suicidal event, but as an active, real-time early warning system integrated into a family care network. Our case thus represents the first report to document this specific role of CGM in the prevention of a potentially fatal insulin overdose.

The patient's subsequent non-adherence to structured inpatient psychiatric care, and opting instead for sporadic private outpatient consultations, reflects a well-documented pattern in BPD, where ambivalence toward treatment and impulsivity frequently undermine sustained engagement. Cocchi et al. reported that Cluster B personality disorders, including BPD, exhibited among the lowest pharmacological adherence rates in a cohort of 200 patients with personality disorders, with only 61.3% achieving positive adherence, driven by impulsivity, emotional dysregulation, and an unstable self-identity [21]. Despite this, she reported subjective improvement and maintained CGM use at one-month follow-up, suggesting that low-threshold, technology-assisted monitoring may serve as a pragmatic adjunct when formal psychiatric adherence is limited. This case reinforces the necessity of flexible, structured outpatient frameworks that account for the specific challenges of BPD, including proactive family involvement, suicide risk screening, and patient education in the context of chronic medical comorbidity such as T2DM.

Finally, the case raises key clinical questions about therapeutic adherence, polypharmacy, and the adequacy of outpatient follow-up in individuals with severe psychiatric and medical comorbidity. Given that suicidality in BPD often manifests in impulsive, ambivalent gestures, frequently involving self-poisoning or overdose​ [22], this case reinforces the necessity of structured mental health follow-up, suicide risk screening, and patient/family education in the context of chronic illnesses such as T2DM. This case report carries inherent limitations. As a single case, findings cannot be generalized to broader populations of patients with diabetes and comorbid psychiatric disorders. The capillary glucose value at emergency department arrival (60 mg/dL) was obtained after oral glucose administration by the patient's daughter before arrival. The pre-intervention glucose value was not independently documented in the clinical record, limiting precise correlation between the CGM trace and clinical severity at the time of discovery. Furthermore, the relative contribution of hypoglycemia versus benzodiazepine toxicity to the patient's somnolence could not be formally quantified. Finally, the patient's non-adherence to inpatient psychiatric care limits conclusions about the long-term effectiveness of the proposed treatment framework.

## Conclusions

This case illustrates how CGM technology, originally designed for metabolic control, can play an unexpected yet critical role in suicide prevention. In patients with coexisting psychiatric disorders and chronic medical conditions such as type 2 diabetes, access to potentially lethal medications such as insulin necessitates heightened vigilance. The integration of CGM systems into family-shared care provided a real-time alert that enabled prompt intervention and likely prevented a fatal outcome. Clinicians should consider the broader applications of digital health tools, particularly in patients with a history of suicidal behavior, and reinforce collaborative care models that include family involvement, structured psychiatric follow-up, and rational pharmacotherapy. As one of the first reports to document the life-saving potential of real-time CGM in this context, this case underscores the need for further research into the integration of digital health technologies within psychiatric risk management frameworks.
